# Supplementary material for: Persistent Parental RNAi in the Beetle Tribolium castaneum Involves Maternal Transmission of Long Double‐Stranded RNA
Source: Adv Genet (Hoboken). 2022 Mar 20;3(3):2100064. doi: 10.1002/ggn2.202100064 (PMC9744488; doi:10.1002/ggn2.202100064)
Supplement: Supplementary file 1 — Supporting Information [file GGN2-3-2100064-s002.pdf]

ADVANCED GENETICS
Open Access

Supporting Information

for *Advanced Genetics*, DOI 10.1002/ggn2.202100064

Persistent Parental RNAi in the Beetle *Tribolium castaneum* Involves Maternal Transmission of Long Double-Stranded RNA

*Thorsten Horn, Kalin D. Narov and Kristen A. Panfilio**

Persistent parental RNAi in the beetle *Tribolium castaneum* involves maternal transmission of long double-stranded RNA

Supporting Information

Figure S1. Additional phylogenies with species subsampling for SID-1/SIL proteins.

Figure S2. Negative correlation of nested RT-qPCR amplicon length and detection of dsRNA.

Table S1. Primers used in this study.

Table S2. Genome assembly versions queried by BLAST.

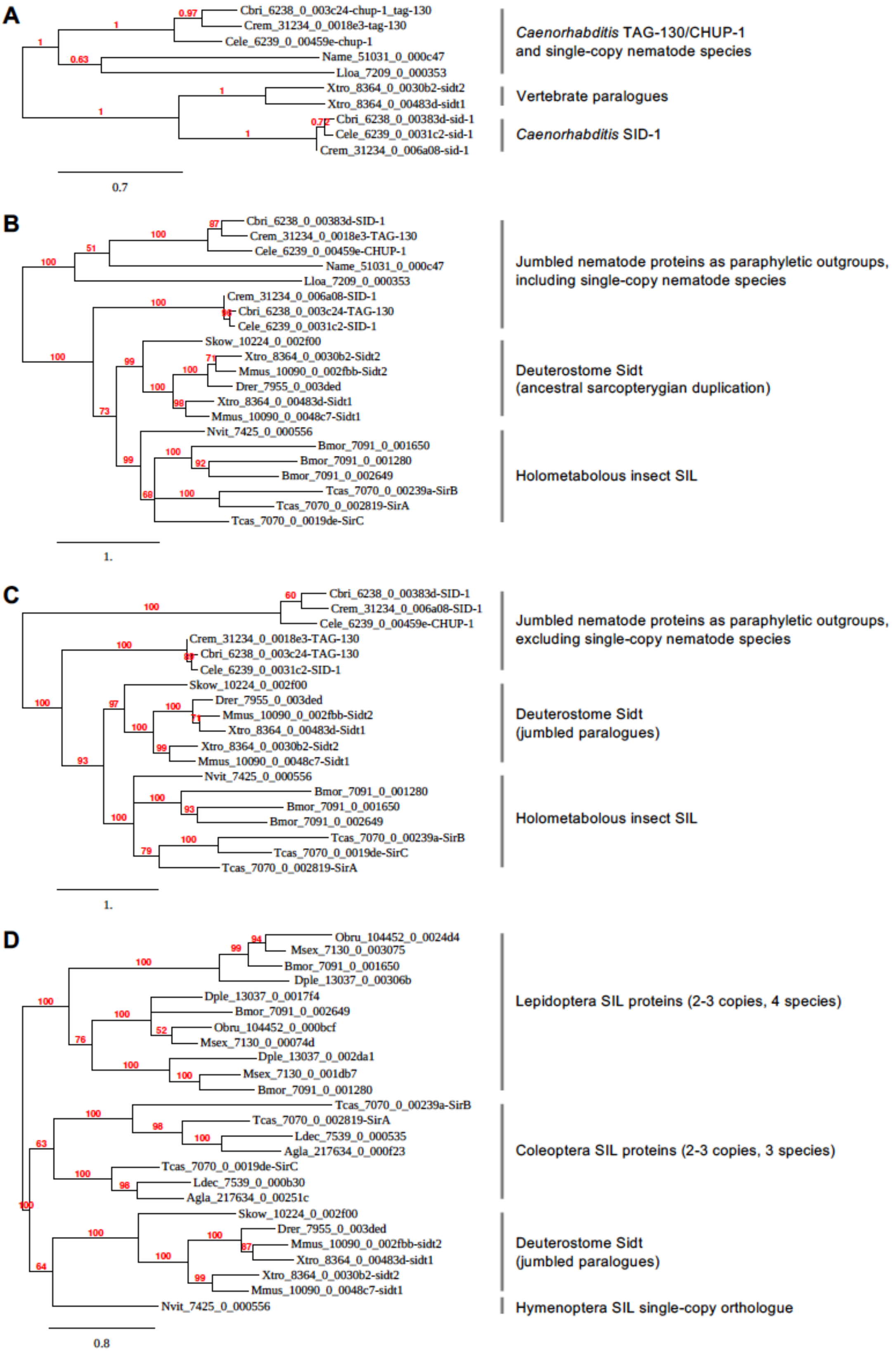

Figure S1. Additional phylogenies with species subsampling for SID-1/SIL proteins. (A-D) Maximum likelihood phylogenies of selected SID-1 homologues. The branch length unit represents substitutions per site. All nodes have ≥50% support. The designation "jumbled" highlights clades that conflate distinct genes (nematode SID-1 with TAG-130/CHUP-1, vertebrate Sidt1 with Sidt2), which did not occur across all trees.

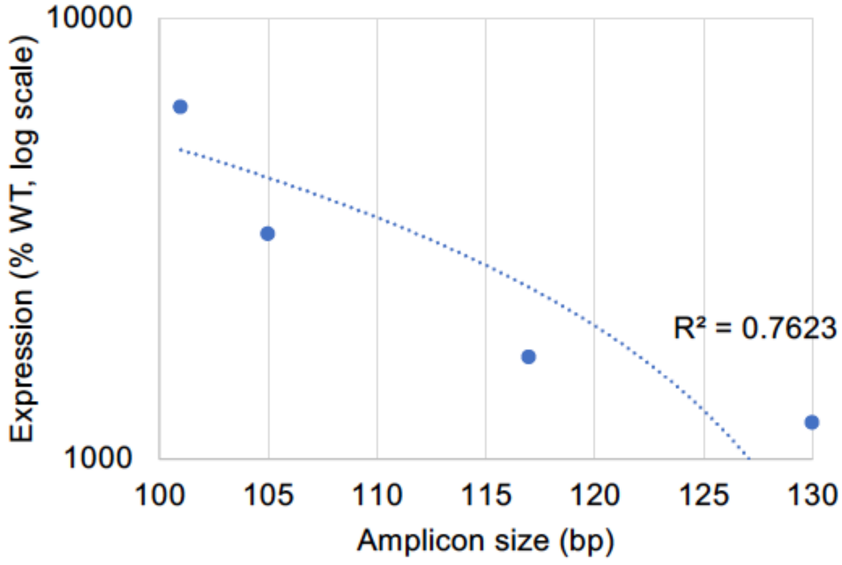

Figure S2. Negative correlation of nested RT-qPCR amplicon length and detection of dsRNA (expression in excess of WT), assayed for *Tc-zen1* at 16-24 h, as in main text Figure 1C. Fragments 3 and 4 are shorter than Fragments 2 and 5. Logarithmic trendline for mean expression level (% WT) *vs*. amplicon length: $R^2 = 0.76$.

Table S1. Primers used in the study. Note that primers for RNAi (dsRNA synthesis) also included an adapter sequence, 5'-GGCCGCGG-3' (forward primers) or 5'-CCCGGGGC-3' (reverse primers), for subsequent amplification with T7 promoter universal primers (adapters not shown in table). The T7 universal primers are: 5'-universal primer 5'-GAGAATTCTAATACGACTCACTATAGGGCCGCGG-3', and 3'-universal primer 5'-AGGGATCCTAATACGACTCACTATAGGGCCCGGGGC-3'.

Application	Gene and fragment ID	Primer direction	Sequence (5' to 3')	Amplicon length (bp)
**RNAi**				
	Tc-zen1 (TC000921)	forward	TCCCAATTTGAAAACCAAGC	688
		reverse	CGTTCCACCCTTCCTGATAA	
	Tc-chs1 (TC014634)	forward (F1)	CACCAGGACTGTGCA	390
		reverse (R1)	GGCTTTTTGGACGAT	
	DsRed2 (EU257621.1)	forward	AGTTCATGCGCTTCAAGGTG	600
		reverse	TGGTGTAGTCCTCGTTGTGG	
	Tc-tup (TC033536), NOF 1	forward (F1)	CGTGCGAGATGGTAAAACCT	306
		reverse (R1)	TTGCTCAAGCTGGTGTTGTT	
	Tc-tup (TC033536), NOF 2	forward (F2)	CACGTTGAGGACGTGCTATG	347
		reverse (R2)	GCTGATGGGGTTGCTCTAAG	
	Tc-gcl (TC001571)	forward (F1)	CGTTGATCAGTGGTGTTGCA	437
		reverse (R1)	TCGCTTCCTCCCAGAAATGT	
**RT-qPCR**				
	Tc-RpS3 (TC008261)	forward	ACCTCGATACACCATAGCAAGC	186
		reverse	ACCGTCGTATTCGTGAATTGAC	
	Tc-zen1 (5'-3'):			
	Fragment 1 outside dsRNA	forward	TCCTGTTGTGAGTCAGTGCA	223
		reverse	CAGTTCCAATCAGAAGGTGGA	
	Fragment 2 inside dsRNA	forward	TGAAAACCAAGCCGTTCTGC	169
		reverse	CAGTTCCAATCAGAAGGTGGA	
	Fragment 3 inside dsRNA	forward	TCCACCTTCTGATTGGAACTG	161
		reverse	CGTTGGGGTTGAGTTTCTTG	
	Fragment 4 inside dsRNA	forward	CGGCCCAATTAGTGGAATTA	101
		reverse	ACGCTCACTCAGGTTCAGGT	
	Fragment 5 inside dsRNA	forward	CCATCGACAGTGCAAACCAA	130
		reverse	TCCTCTTGTTTGGGCAAAGC	
	Fragment 6 outside dsRNA	forward	CCATCGACAGTGCAAACCAA	190
		reverse	GTTAAAGCAGGCTGGGACAC	
	Tc-chs1 **(3')**	forward	ATTCTGTAACCGGGACCTGG	
		reverse inside dsRNA	CCAGAAGGCGAAGATCAAGC	100
		reverse outside dsRNA	ATGAGGAAGTGGGAGAAGGC	186
	DsRed **(5')**	forward inside dsRNA	AGTTCATGCGCTTCAAGGTG	123
		forward outside dsRNA	GCTCCTCCAAGAACGTCATC	147
		reverse	CCTTGGTCACCTTCAGCTTC	

Table S2. Genome assembly versions queried by BLAST. These resources were interrogated with tBLASTn queries for selected SID-1 proteins (see main text Figure 5B). Accessed at the i5K@NAL site, most recent access date: 13 October 2021.

**Taxonomic grouping**	**Species**	**Species abbrevi-ation**	**Assembly version**
Paraneoptera > Hemiptera > Sternorrhyncha	*Bemisia tabaci*	Btab	Genome Assembly - Bemisia tabaci genome assembly GCF_001854935.1 (ASM185493v1)
Paraneoptera > Hemiptera > Sternorrhyncha	*Diaphorina citri*	Dcit	Genome Assembly - NCBI-diaci1.1 (Current RefSeq assembly version)
Paraneoptera > Hemiptera > Heteroptera	*Gerris buenoi*	Gbue	Genome Assembly - Gbue.scaffolds.50_new_ids.fa
Paraneoptera > Hemiptera > Heteroptera	*Cimex lectularius*	Clec	Genome Assembly - Clec_Bbug02212013.genome_new_ids.fa
Paraneoptera > Hemiptera > Heteroptera	*Halyomorpha halys*	Hhal	Genome Assembly - Halyomorpha halys genome assembly GCA_000696795.3
Paraneoptera > Hemiptera > Heteroptera	*Oncopeltus fasciatus*	Ofas	Genome Assembly - Ofas.scaffolds_new_ids.fa
Paraneoptera > Thysanoptera	*Frankliniella occidentalis*	Focc	Genome Assembly - Frankliniella occidentalis genome assembly GCA_000697945.4
Holometabola > Hymenoptera	*Athalia rosae*	Aros	Genome Assembly - Aros01112013-genome_new_ids.fa
Holometabola > Coleoptera	*Anoplophora glabripennis*	Agla	Genome Assembly - Agla_Btl03082013.genome_new_ids.fa
Holometabola > Coleoptera	*Leptinotarsa decemlineata*	Ldec	Genome Assembly - Leptinotarsa decemlineata genome assembly GCF_000500325.1
Holometabola > Coleoptera	*Tribolium castaneum*	Tcas	Genome Assembly - Tribolium castaneum genome assembly Tcas5.2 (GCF_000002335.3), genomic scaffolds
Holometabola > Diptera	*Drosophila biarmipes*	Dbia	Genome Assembly - Drosophila biarmipes genome assembly GCF_000233415.1
Holometabola > Diptera	*Drosophila elegans*	Dele	Genome Assembly - Drosophila elegans genome assembly, ASM1815250v1
Holometabola > Diptera	*Drosophila kikkawai*	Dkik	Genome Assembly - Drosophila kikkawai genome assembly, ASM1815253v1
